# Pharmacologic Vitreolysis

**Published:** 2010-01

**Authors:** Hossein Nazari, Mehdi Modarres-Zadeh, Arash Maleki

**Affiliations:** Eye Research Center, Iran University of Medical Sciences, Tehran, Iran

**Keywords:** Pharmacologic Vitreolysis, Enzymatic Vitreolysis

## Abstract

The vitreoretinal interface is involved in a wide range of vitreoretinal disorders and separation of the posterior vitreous face from the retinal surface is an essential part of vitrectomy surgeries. A diverse range of enzymatic and non-enzymatic agents are being studied as an adjunct before or during vitrectomy to facilitate the induction of posterior vitreous detachment. There is a significant body of knowledge in the literature about different vitreolytic agents under investigation for a variety of pathologies involving the vitreoretinal interface which will be summarized in this review.

## INTRODUCTION

**Figure 1:**
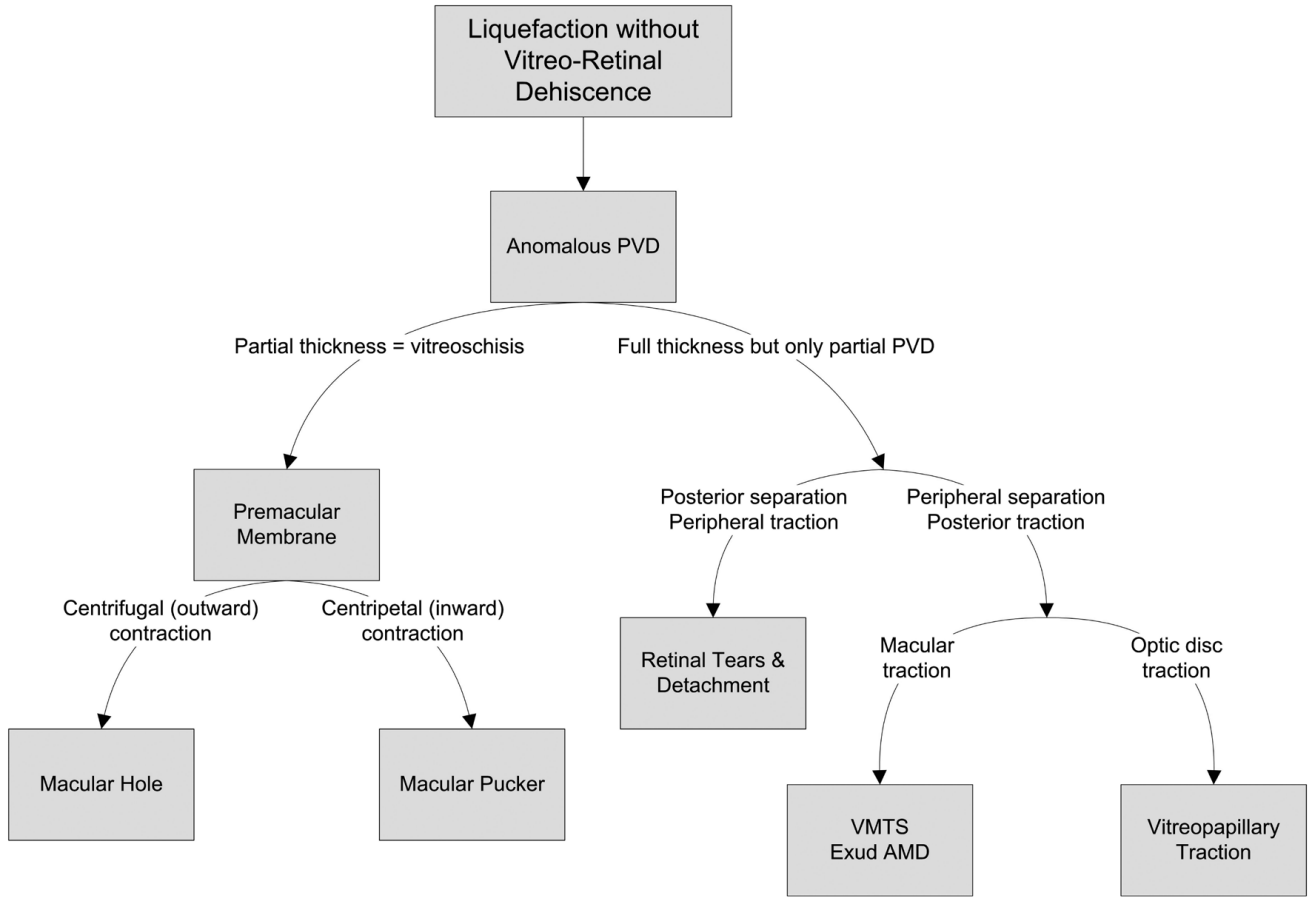
Diagram summarizing different pathologies arising from anomalous posterior vitreous detachment (PVD). VMTS, vitreomacular traction syndrome; Exud: exudative; AMD, age-related macular degeneration

The dynamic interaction between the peripheral cortical vitreous and the retina is believed to play a significant role in different physiologic and pathologic processes. The exact mechanism of attachment between the cortical vitreous and the internal limiting membrane (ILM) is not fully understood, but it is believed that connecting protein molecules are responsible for the adhesion between vitreous collagen fibrils and the ILM. The vitreous cortex is more adherent to the retina where the ILM is thinner such as the vitreous base, optic nerve head, along major vascular arcades and around the fovea. The ILM increases in thickness with aging which may explain why posterior vitreous detachment (PVD) occurs more frequently in older subjects.^1^ PVD is characterized by vitreous liquefaction and vitreoretinal separation.^2^ Incomplete PVD or anomalous vitreoretinal adhesions are the basic mechanisms in a variety of vitreoretinal pathologies (Fig. 1).^3^ On the other hand, there is increasing evidence that complete detachment of the vitreous may protect against a variety of disorders such as diabetic retinopathy^4^ and exudative age-related macular degeneration (AMD).^5^ Apparently different disorders may follow a common initiating event i.e. anomalous PVD (Fig. 1).

During vitrectomy surgery in eyes without PVD, the posterior hyaloid face is mechanically separated from the ILM and the optic nerve head by vitreous pic or applying passive or active suction.^6^ These maneuvers may be associated with complications such as iatrogenic retinal tears, nerve fiber layer damage and even retinal detachment.^7^ Complications of mechanical PVD induction are more prevalent in eyes with stronger vitreoretinal adhesions such as that of young patients. Alternative methods for induction of PVD have been investigated extensively among which pharmacologic separation of vitreoretinal adhesions has long been an attractive idea for researchers and clinicians.

Agents used for pharmacologic vitreolysis can be categorized as “enzymatic” or “nonenzymatic” according to their mechanism of action. The majority of proposed agents for pharmacologic vitreolysis are enzymes which include but are not limited to tissue plasminogen activator (tPA),^8^ plasmin,^9^ microplasmin,^10^ nattokinase,^11^ chondroitinase,^12^ dispase,^13^ and hyaluronidase.^14^ Nonenzymatic agents used for induction of PVD include urea/vitreosolve and Arginine-Glycine-Aspartate peptides.^3^

Herein, we will briefly review the literature related to pharmacologic vitreolysis. A Pubmed search by key words “vitreolysis”, “pharmacologic vitreolysis”, “enzymatic vitreolysis”, “plasmin and vitrectomy”, and “microplasmin and vitrectomy” was performed and full text English articles were retrieved.

## CLASSIFICATION OF VITREOLYTIC AGENTS

In a classification system proposed by Sebag,^3^ vitreolytic agents have been categorized on the basis of their biologic effect into those which induce vitreous liquefaction (“liquefactants”) and those which induce dehiscence at the vitreoretinal interface (“interfactants”). Table 1 classifies different enzymatic and nonenzymatic agents on the basis of their major biologic activity.^3^

### Tissue Plasminogen Activator (tPA)

Tissue plasminogen activator (tPA) is a serine protease involved in conversion of plasminogen to plasmin, the main enzyme responsible for blood clot lysis. In a randomized study, Hesse et al^15^ compared intravitreal injection of 25 μg tPA to balanced salt solution (BSS) before vitrectomy in 10 patients with proliferative diabetic retinopathy (PDR). They concluded that tPA caused disintegration of the vitreoretinal interface by posterior vitreous detachment, facilitating pars plana vitrectomy (PPV) without severe side effects. They also reported on injecting 25 μg of recombinant tPA in rabbit eyes. All tPA treated eyes showed vitreous cortex separation from the retina and the posterior lens surface one week after injection, while none of the control eyes showed PVD.^8^ They suggested that this method may be useful before mechanical vitrectomy.^8^ Although intravitreal tPA injection has previously been reported for management of subretinal hemorrhage in several articles, complications such as vitreous hemorrhage which are related to its fibrinolytic activity have been a main concern.^16^,^17^ Currently, intraocular tPA is mainly used for treatment of postvitrectomy fibrin formation and submacular hemorrhage but has not been shown to be safe and/or effective for other indications such as induction of PVD during or before vitrectomy.^18^

### Plasmin

Plasmin is a nonspecific protease which plays an active role in multiple biologic processes such as fibrinolysis, neovascularization and activation of other enzymes such as matrix metalloproteinases (MMP).^19^ Furthermore, plasmin acts directly on fibronectin and laminin, two major proteins responsible for adhesion between the posterior hyaloid face and the ILM.^9^,^20^ Induction of PVD by plasmin has been accomplished in several animal models.^9^,^19^–^24^ Electron microscopic and electrophysiologic studies have shown no evidence of retinal toxicity with intravitreal injection of plasmin.^21^,^24^ In addition to PVD induction, plasmin liquefies the vitreous; the combination of these two effects along with its safety profile, make plasmin a suitable agent for pharmacologic vitreolysis. Intravitreal plasmin injection before or during vitrectomy has been studied in multiple case series for diabetic macular edema associated with a thick and adherent posterior hyaloid face,^25^ idiopathic and traumatic macular holes,^26^–^28^ proliferative diabetic retinopathy,^29^ diabetic tractional retinal detachment,^30^ vitreomacular traction syndrome^31^ and retinopathy of prematurity.^32^,^33^

Azzolini et al^25^ evaluated the intraoperative use of plasmin for induction of PVD in eyes with diabetic macular edema associated with posterior vitreous cortex contraction. A volume of 0.1 to 0.2 ml containing 0.8 to 1.2 IU of autologous plasmin was injected into the vitreous body 25 minutes before surgery. In 4 out of 12 plasmin treated eyes versus one out of 10 control eyes, partial or complete PVD was detected during vitrectomy. They found no significant difference in final retinal thickness, but plasmin treated eyes had significantly better visual acuity. They concluded that autologous plasmin may be useful for induction of PVD and facilitation of vitrectomy surgery.

Asami et al^34^ injected autologous plasmin before vitrectomy in 10 eyes with diabetic macular edema without PVD and compared them with 10 similar controls. They performed ILM peeling during surgery and evaluated the specimens with transmission electron microscopy. In the plasmin treated group, 8 eyes showed a smooth surface on the vitreous side of the ILM and only sparse vitreous remnants were seen in two other eyes. In contrast only 3 control eyes showed a smooth ILM surface. This study along with other similar reports showed that remnants of vitreous strands are more effectively eliminated from the inner surface of the ILM with plasmin assisted vitrectomy.^34^–^36^

There are several studies evaluating autologous plasmin for macular hole surgery. Although there is no randomized clinical trial comparing macular hole closure rates and complications of vitrectomy with and without plasmin, multiple small case series suggest that plasmin facilitates vitreous surgery.^26^–^28^,^37^ In a retrospective study, Wu et al^27^ reported the results of autologous plasmin enzyme assisted vitrectomy on 13 pediatric patients aged 1–15 years with a traumatic macular hole. The macular hole was closed successfully in 12 cases and they proposed that autologous plasmin may be a helpful adjunct during vitrectomy for traumatic macular holes.

Hirata and colleagues^29^ evaluated the feasibility of autologous plasmin for surgical treatment in six patients with bilateral PDR. They used autologous plasmin in one eye of each patient and compared the fellow eyes in terms of surgical time and incidence of retinal tears. Both surgical time (68 versus 89 minutes, P=0.04) and incidence of retinal tear (nil versus 3 eyes) were significantly lower in the plasmin group. Moreover, no additional surgical procedures for removing the proliferative membrane including delamination or segmentation were needed in the plasmin group. However, there was no significant difference between the two groups regarding final visual outcomes.

Tsukahara et al^32^ reported six consecutive eyes of four premature infants with stage 5 retinopathy of prematurity (ROP). Four eyes showed retinal detachment with closed funnel configuration. After lensectomy and dissection of anterior proliferative membranes, 0.03 to 0.22 IU autologous plasmin was administered into the vitreous cavity. Fifteen to 30 minutes later, the proliferative membranes were successfully removed without causing iatrogenic breaks in all eyes. Complete reattachment of the posterior pole was achieved in all six eyes. No obvious complication was observed during the follow-up period, which ranged from 11 to 14 months.

In a large case series reported by Wu et al,^33^ 80 eyes with stage 5 ROP underwent vitreoretinal surgery using autologous or maternal plasmin. Fifty-three eyes had previously undergone vitrectomy with or without retinal breaks. Following surgery for all 80 eyes, anatomic success was achieved in 68.8% of the cases, in contrast to 23% to 59% in previous reports. The authors concluded that plasmin-assisted vitrectomy in eyes with advanced ROP with or without previous vitrectomy increases the chance of anatomical and visual improvement.

Diaz-Llopis and colleagues^38^ reported their experience with low dose intravitreal autologous plasmin injection in lieu of vitrectomy for treatment of patients with refractory diabetic macular edema. Intravitreal plasmin was injected in one eye of 16 patients with refractory diffuse diabetic macular edema and the fellow eyes served as controls. One month after injection, central macular thickness (CMT) was reduced and best-corrected visual acuity (BCVA) increased significantly in plasmin injected eyes. BCVA and CMT were stable up to 6 months. The authors proposed that intravitreal plasmin injection effectively reduces macular thickness and improves vision in diabetic patients with macular edema refractory to conventional laser treatment. They did not report the condition of the vitreoretinal interface in their patients.

In most of the above-mentioned reports, plasmin was derived preoperatively from the patient’s blood. The most commonly used technique is affinity chromatography. Plasminogen obtained by this technique is concentrated and converted by a plasminogen activator to plasmin. The plasmin should pass sterility controls and be refrigerated prior to use.^39^ This technique is time consuming, cumbersome and difficult. An easier technique has been described which involves centrifuging autologous blood for separating plasminogen containing plasma followed by activating plasminogen with streptokinase or urokinase.^40^ Plasmin is not commercially available for clinical use as mentioned earlier, furthermore enzyme activity is dependent on several variables including plasminogen level in the blood. Therefore plasmin has not gained widespread use for vitrectomy surgery.^41^

### Microplasmin

The development of recombinant microplasmin solved most of the problems of autologous plasmin. Microplasmin contains only the enzymatic portion of the plasmin molecule while the other domains are omitted.^42^ This makes the microplasmin molecule much smaller than the original molecule (28 KD vs 80 KD) with the same enzymatic activity. Microplasmin has been shown to induce vitreous liquefaction and PVD without any evidence of retinal toxicity.^10^ Sakuma et al^10^ injected different doses of microplasmin into rabbit eyes and showed that intravitreal injection of microplasmin causes no electroretinographic or ultrastructural retinal abnormalities. Doses greater than 125 μg induced complete PVD. The authors suggested that microplasmin may be a useful adjunct to vitreous surgery or could be used to induce PVD without vitreous surgery. Chen et al^42^ have also shown that microplasmin is able to induce PVD without retinal toxicity in rabbit eyes.

de Smet and colleagues^43^ have recently shown that PVD induction by microplasmin is both dose and time dependent in porcine eyes. One hour after injection, the posterior pole was detached in 100% of the eyes with 125 μg of microplasmin but the midperipheral vitreous was also detached using the 250 μg dosage. Vitreous at the ora however, was not detached. After 2 hours of exposure, midperipheral detachment was observed with the 125 μg dose as well. A smooth retinal surface was seen at the site of enzyme induced PVD. They found the minimum effective dose to be 125 μg.

In a recent animal study, Sebag et al^44^ reported that pharmacologic vitreolysis with recombinant microplasmin increased vitreous diffusion coefficient as determined with dynamic light scattering. This could result in metabolic changes in the retina. Quiram and colleagues^45^ recently reported that PVD induction by microplasmin increases oxygen level in the vitreous cavity.

de Smet et al^46^ have published the results of MIVI I (Microplasmin for Intra Vitreous Injection) trial. In this multicenter, prospective, uncontrolled, dose-escalation, phase I/II clinical trial, the safety and preliminary efficacy of 4 doses and several exposure times of intravitreal microplasmin given before vitrectomy for vitreomacular traction maculopathy were evaluated. A single intravitreal injection of 4 different doses (25, 50, 75, or 125 μg in 100 μl) of microplasmin was administered 1 to 2 hours, 24 hours, or 7 days before planned pars plana vitrectomy. Increasing doses and incubation times resulted in an increased rate of PVD in some patients. They proposed that although the drug is well tolerated and capable of inducing pharmacologic PVD in some patients, larger, controlled trials are warranted. At the present, ThromboGenics, a Belgium based pharmaceutical company, provides microplasmin commercially.

### Nattokinase

Nattokinase, a strong fibrinolytic agent, was originally derived from natto which is a popular soybean cheese in Japan.^11^ Nattokinase (subtilisin NAT) is a serine protease composed of 275 amino acids produced by Bacillus subtilis (natto). It can be administered orally and is present in foods prepared from fermented soybean. Because of its potent fibrinolytic effect it is now under investigation for prevention of cardiovascular thrombotic events and reducing blood pressure.^47^,^48^ Takano et al^11^ injected 0.1 and 1 fibrin-degradation unit (FU) of nattokinase into rabbit eyes and showed that both doses could produce complete PVD with a smooth inner ILM surface, but the higher dose (1 FU) was associated with some retinal toxicity. More investigations are needed to clarify the safety and efficacy aspects of this new vitreolytic agent.

### Chondroitinase

Hermel and Schrage^49^ investigated the efficacy of human and porcine plasmin and chondroitinase in creating vitreoretinal separation in the pig eye. Chondroitinase (1 U) failed to increase PVD rates in pig eyes after 1 hour of incubation, but spontaneous PVD occurred more frequently and to a larger extent in plasmin treated eyes than placebo controls. Chondroitinase also failed to produce any effect on the vitreoretinal interface but in plasmin-treated eyes, scanning electron microscopy showed a smooth ILM surface free from vitreous remnants. Staubach et al^50^ injected plasmin, hyaluronidase and chondroitinase into freshly enucleated pig eyes. All enzymes showed increased vitreous removal rates, however chondroitinase was the weakest.

### Dispase

Dispase is a protease which was studied for induction of PVD in one of the earliest animal studies in this field. Tezel et al^51^ injected dispase into human and porcine cadaver eyes. After 15 minutes of incubation, partial or total PVD was seen in the majority of enucleated porcine and human eyes. Microscopic examination demonstrated that dispase cleaved the attachment between the posterior hyaloid and the ILM with minimal damage to the inner retina. Retinal cell viability and the mechanical properties of the retina were similar in dispase-treated and control eyes. They concluded that the enzyme may be useful in removing cortical vitreous during vitreous surgery. While some studies have shown that dispase is useful for PVD induction,^52^ most experimental studies have indicated some harmful effects.^53^–^55^

Dispase is a proteolytic enzyme and dissociates tissues, therefore it has been suggested that intraocular injection of dispase could trigger events leading to proliferative vitreoretinopathy (PVR). Frenzel et al^56^ injected dispase into rabbit eyes and showed that it initiated the development of PVR without addition of exogenous cells, growth factors, or cytokines typically found in PVR membranes. A cascade of events was probably triggered by dispase, causing native cells and factors to produce PVR. Other investigators have shown that intravitreal dispase can result in cataracts and lens subluxation as well as reproducible induction of PVR.^57^

### Hyaluronidase

While other vitreolytic agents act by separating the vitreoretinal interface, hyaluronidase exerts its effect by dissolving the glycosaminoglycan network of the vitreous gel which is predominantly composed of hyaluronan. Collagen fibrils of the vitreous are very resistant to proteolytic degradation and therefore not a good target for pharmacological vitreolysis, but enzymatic degradation of the hyaluronan helps vitreous degradation and thus may facilitate dispersal of vitreous hemorrhage.^58^ Some studies has shown that intravitreal injection of hyaluronidase could increase the rate of vitreous removal during vitrectomy.^50^

Highly purified bovine hyaluronidase (Vitrase) is the only vitreolytic agent that has passed phase III trials.^59^ Hyaluronidase basically acts on the glycoside bonds of hyaluronan and other mucopolysaccharides in the vitreous and causes vitreous liquefaction.^20^ There is no evidence to support a direct effect of hyaluronidase on the vitreoretinal interface. Vitrase was evaluated in two phase III trials for management of vitreous hemorrhage.^60^

A total of 1,125 patients were randomized in a 1:1:1 ratio to intravitreal injection of saline and 55 IU or 75 IU of hyaluronidase. The primary end point was clearance of vitreous hemorrhage. There was a statistically significant improvement in the 55 IU group at 1, 2 and 3 months as compared to the saline group. Intravitreal injection of hyaluronidase was well tolerated without any significant complication.^60^ Although the study investigators concluded that intravitreal hyaluronidase was useful in the management of vitreous hemorrhage, this agent has not yet received FDA approval for this indication. Furthermore, other studies have shown that although hyaluronidase may help in vitreous liquefaction and vitreous hemorrhage clearance, it does not induce vitreoretinal separation.^20^

Zhi-Liang and colleagues^61^ evaluated intravitreal injection of plasmin, hyaluronidase, combined plasmin and hyaluronidase, and BSS to induce PVD in diabetic rat eyes. Hyaluronidase alone was ineffective, whereas plasmin alone induced partial PVD, a potentially dangerous condition for diabetic eyes. However, the combination of hyaluronidase and plasmin induced complete PVD in diabetic rats. PVD induction was more difficult in diabetic rats than in healthy rats. Wang et al^20^ showed that vitreous injection of plasmin and hyaluronidase induced complete PVD with no obvious toxicity; plasmin induced partial PVD, but hyaluronidase had no effect. It has also been commented that the combination of plasmin and hyaluronidase is not necessary and an appropriate dose of intravitreal plasmin alone can induce complete PVD.^62^

## FUTURE HORIZONS

Sutureless vitrectomy using the 23 or 25 gauge systems has gained increasing popularity in recent years. One of the main concerns in small gauge vitrectomy is the decreased rate of vitreous removal by smaller vitrectomy probes. Hermel and associates^63^ measured the volume of vitreous removed by the vitreous cutter at pre-specified time intervals in rabbit eyes with and without plasmin incubation. They found that intravitreal plasmin injection could increase the rate of vitreous removal which showed a linear correlation with incubation time. It may be concluded from this study, as well as those mentioned earlier, that vitreolytic agents may facilitate vitrectomy and reduce operative time.

Enzyme-induced PVD in animal models increases lens nucleus oxygen levels. There may also be some correlation between the presence of PVD and the rate of nuclear cataract progression in humans.^64^ The effect of vitreolytic agents in the progression of cataract needs to be elaborated. Pharmacologic PVD induction and the ensuing increased vitreous oxygen levels may also have some effect on AMD and retinal ischemic diseases.^44^,^45^ The possible role of plasmin in stimulation of PVR should be further evaluated. If this is proven to be the case, antiplasmin agents may have a role in the future management of PVR.^65^

## CONCLUSION

Creating a complete and atraumatic vitreoretinal separation and attaining an ultrastructurally smooth and vitreous-free ILM surface is an essential step for successful vitreoretinal surgery. This may also improve retinal oxygenation and decrease the risk of PVR. Although multiple agents have been studied, recent reports on the beneficial effects of plasmin and microplasmin have raised strong hopes that pharmacologic vitreolysis may eventually find its way to clinical practice. Further investigations will demonstrate whether enzymatic vitreolysis could be used as an adjunct and/or alternative treatment for treatment of vitreoretinal disorders.

## Figures and Tables

**Table 1:** Classification of pharmacologic vitreolytic agents proposed by Sebag^3^

Pharmacologic vitreolysis classification based on biologic activity
**Liquefactants** (agents that liquefy the gel vitreous)
Nonspecific: tPA, plasmin, microplasmin, nattokinase, vitreosolve*
Substrate specific: chondroitinase, hyaluronidase
**Interfactants** (agents that alter the vitreoretinal interface)
Nonspecific: tPA, plasmin, microplasmin, nattokinase, vitreosolve*
Substrate specific: dispase, chondroitinase
RGD-peptides*
tPA, plasmin, microplasmin, nattokinase, and vitreosolve are believed to be both liquefactants and interfactants.

* Nonenzymatic agent.

tPA, tissue plasminogen activator; RGD, Arginine-Glycine-Aspartate.
